# Tumor Lysis Syndrome in Patients With Hepatocellular Carcinoma: A Systematic Review of Published Case Reports

**DOI:** 10.7759/cureus.19128

**Published:** 2021-10-29

**Authors:** Jen-Wei Chou, Ken-Sheng Cheng, Trupti Akella, Chi Chan Lee, Teressa Ju

**Affiliations:** 1 Gastroenterology, China Medical University Hospital, Taichung, TWN; 2 Gastroenterology, Aventura Hospital & Medical Center, Aventura, USA; 3 Critical Care, Guam Regional Medical Center, Guam, USA; 4 Internal Medicine, NewYork-Presbyterian Queens, New York, USA

**Keywords:** transcatheter arterial chemoembolization (tace), hepatocellular carcinomas (hcc), tumour lysis syndrome (tls), sorafenib, trans arterial chemo embolizations

## Abstract

Tumor lysis syndrome (TLS) is a life-threatening oncologic emergency. It is characterized by massive tumor cell death leading to metabolic derangements and multiple organ failure. It is a rare complication of hepatocellular carcinoma (HCC) with only a few cases have been reported in the literature to date.

We collected and summarized published case reports of tumor lysis syndrome in patients with HCC. We also reported one additional case who developed TLS after sorafenib therapy and wrote a clinical vignette. A comprehensive and current search for relevant articles was conducted in Medline and EMbase through May 2018. A systematic review was performed following the guideline of Preferred Reporting Items for Systematic Reviews and Meta-Analyses (PRISMA).

A total of 28 cases of TLS associated with HCC were enrolled in our review. The median age of included cases was 55.5 years with a male to female ratio of 25:3. The two most common attributed factors of TLS were transcatheter arterial chemoembolization (TACE) (12 cases, 42.9 %) and sorafenib (nine cases, 32.1%). Among enrolled cases, the diameter of the largest tumor was 12 cm. Regarding Barcelona Clinic Liver Cancer (BCLC) staging, seven cases were at least stage A (22.6%), 11 cases were at least stage B (35.5%), and 10 cases were at least stage C (32.3%). The median time of onset of TLS was three days. As for uric acid-lowering agents, nine cases (32.1%) used allopurinol and four cases (14.3%) used rasburicase. Ten cases (35.7%) did not specify the medication prescribed. The overall mortality rate of this cohort was 67.9%.

Compared with patients developing TLS following TACE, patients who had TLS following sorafenib therapy had a later onset of TLS (two days versus seven days, p < 0.001) and a more advanced stage of HCC (p = 0.002). There was a trend toward increased mortality of patients in the sorafenib group in comparison with those in the TACE group (77.8% versus 41.7%, p = 0.18).

The results of this current review suggest that TLS rarely occurs in HCC but carries significantly higher mortality compared to TLS occurring in hematologic malignancies. It may occur shortly after TACE or with a delayed onset following sorafenib therapy. Considering the kaleidoscope of novel therapies and diverse pathogenesis of HCC, it is crucial for clinicians to recognize the clinicolaboratory derangements suggestive of TLS and initiate appropriate management. The present review highlights the need for clinicians to consider TLS within differentials when caring for patients with HCC.

## Introduction and background

Tumor lysis syndrome (TLS) is a life-threatening oncologic emergency. It results from the rapid destruction of tumor cells leading to the efflux of intracellular contents. This, in turn, leads to serum electrolyte imbalance such as hyperuricemia, hyperkalemia, hyperphosphatemia, hypocalcemia, and uremia [1]. Uric acid and calcium phosphate crystals precipitating in renal tubules may cause acute kidney injury (AKI) [1,2]. As a result, excessive metabolites can overwhelm normal homeostatic mechanisms and result in cardiac arrhythmia, neurologic toxicity, or death.

TLS usually occurs in hematologic tumors with high proliferative rates and high tumor burdens such as acute lymphoblastic leukemia and Burkitt's lymphoma [2]. Infrequently, solid tumors with high proliferative rates or with good response to chemotherapy could be complicated by TLS following treatment. Case reports of TLS from different origins of solid tumors have been reported in the literature [3]. The association between hepatocellular carcinoma (HCC) and TLS, however, has rarely been studied. Potential risk factors, prognoses, and clinical courses of this topic remain largely unknown. To our knowledge, clinical data on TLS in patients with HCC is limited to individual case reports, and no systematic review has been performed to analyze this phenomenon in totality. Thus, the present review aims to collect and summarize data of TLS associated with HCC and elucidate the disease characteristics in an organized manner. We will start with a clinical vignette of TLS in HCC and perform a systematic review of published literature.

## Review

A clinical vignette: tumor lysis syndrome in a patient with hepatocellular carcinoma following sorafenib treatment

**Figure 1:**
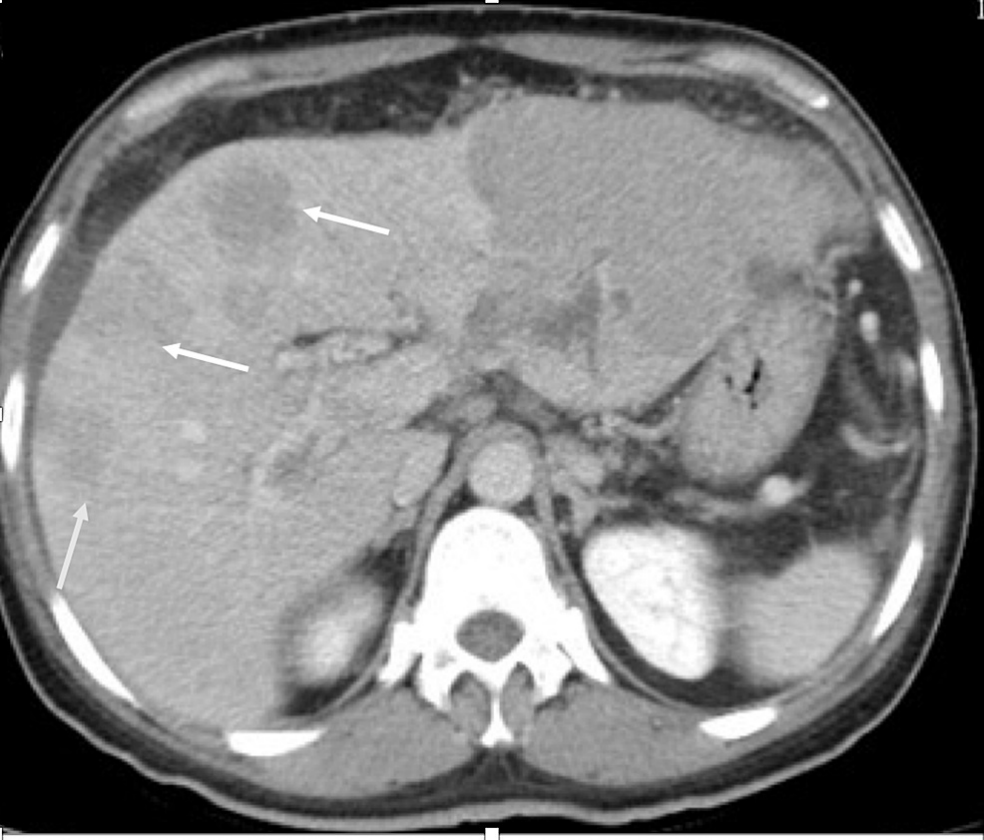
Abdomen CT scan: axial view White arrow: Multiple hypervascular tumors measuring up to 6 cm in size

**Figure 2:**
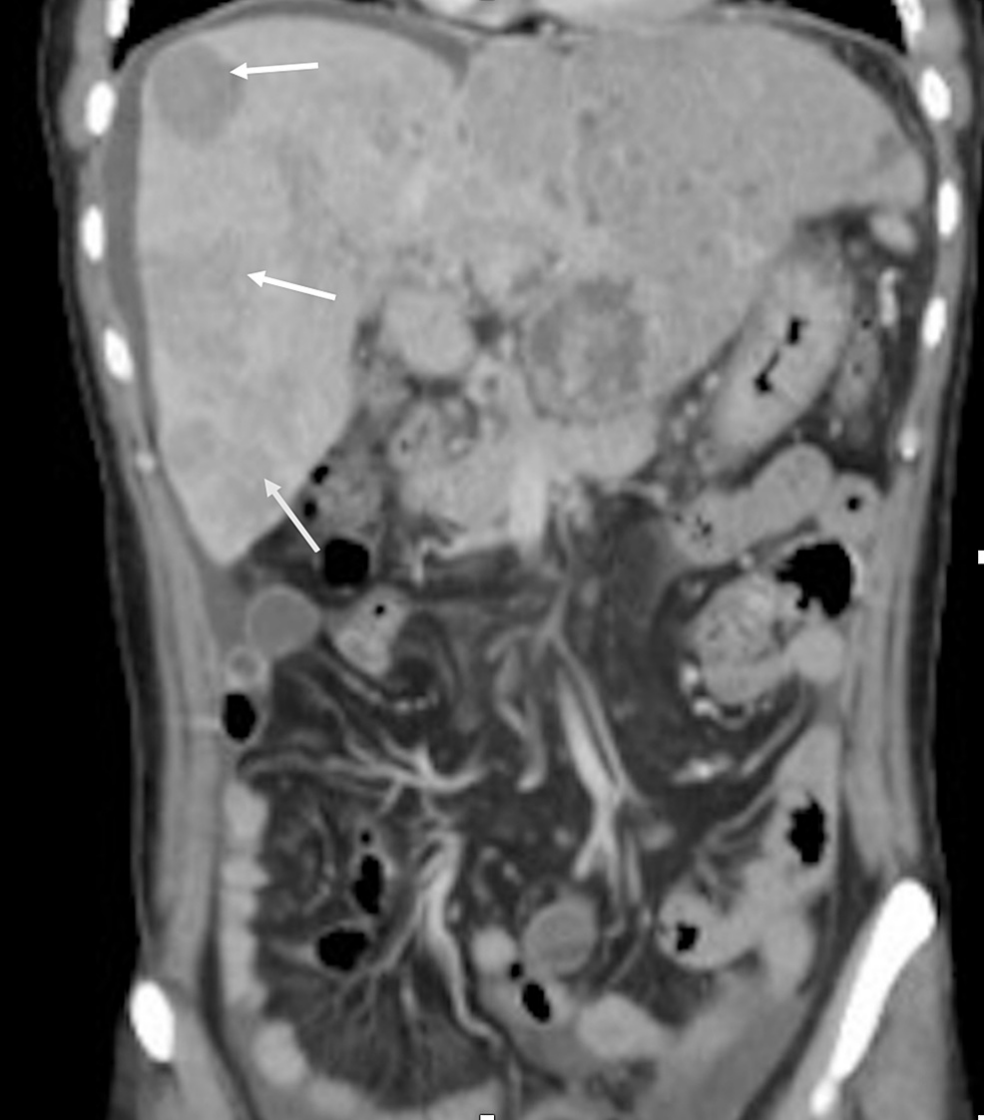
Abdomen CT scan: coronal view White arrow: Multiple hypervascular tumors measuring up to 6 cm in size

A 42-year-old non-alcoholic Asian man was admitted to our hospital because of unintentional weight loss of 5 kg and abdominal pain for one month. He denied vomiting, nausea, fever, or black stool. He was a hepatitis B virus (HBV) carrier because of vertical transmission. His family history was significant for maternal history of liver cirrhosis and HBV leading to death at the age of 50. Upon examination, the patient was not in acute distress and vital signs were within normal range. His conjunctivae were pink, and his sclerae were anicteric. Abdominal examination revealed a soft abdomen without tenderness and an impalpable liver and spleen. Esophagogastroduodenoscopy performed a month before admission revealed esophageal varices, gastric ulcers, and portal hypertensive gastropathy. A proton pump inhibitor was prescribed. Laboratory tests revealed the following: α fetoprotein level > 54000 ng/mL (normal range, < 0.9 ng/mL), aspartate aminotransferase 93 IU/L (5-34), alanine aminotransferase 51 IU/L (0-40), and creatinine 0.96 mg/dL (0.5-1.3). Abdominal USG and CT showed multiple hypervascular tumors, measuring up to 6 cm in size, with portal vein thrombosis and ascites (Figure 1, Figure 2).

Enlarged lymph nodes in the abdomen and multiple nodules over bilateral lung fields were also identified. A diagnosis of hepatocellular carcinoma, imaging staged pT4N1M1 was made. After admission, the patient was treated with tenofovir 300 mg per day and sorafenib 400 mg per day. On the fifth hospital day, the patient began to have a fever with chills and dyspnea. Intravenous flomoxef was prescribed under the impression of intra-abdominal infection. Three days later, the patient developed oliguria (100 mL in 24 hours). The laboratory tests showed the following: calculated glomerular filtration rate: 58 mL/min/1.73㎡ (> 90), uric acid: 12.4 mg/dL (2.5-7.5), lactate dehydrogenase (LDH): 12738 IU/L (98-192), albumin: 3.2 g/dL (3.8-5.3), calcium: 7.4 mg/dL (8.5-10.5), sodium: 126 mmol/L (135-147), potassium: 4.9 mmol/L (3.5-4.9), and creatinine: 1.35 mg/dL (0.9-1.3). A diagnosis of AKI due to tumor lysis syndrome was made. We decided to hold his sorafenib treatment and began aggressive intravenous hydration. We also administered diuretics, urine alkalization, and correction of electrolyte imbalance. Biochemistry profiles, uric acid, LDH levels, and urine output were closely monitored. Feboxustat 80mg/day was prescribed to decrease serum uric acid levels. Two weeks after admission, a drop in hemoglobin was noted. Physical examination revealed an increasingly distended abdomen with bruising of his flanks (Grey Turner's sign). Abdominal paracentesis revealed bloody ascites. Tumor rupture with internal bleeding was suspected, and blood transfusions were begun. The patient refused further aggressive interventional treatment. One month after the start of sorafenib therapy, the patient’s condition deteriorated and he expired due to disease progression.

[A waiver for informed consent was applied to the case vignette. The patient information was de-identified.]

Method

**Figure 3:**
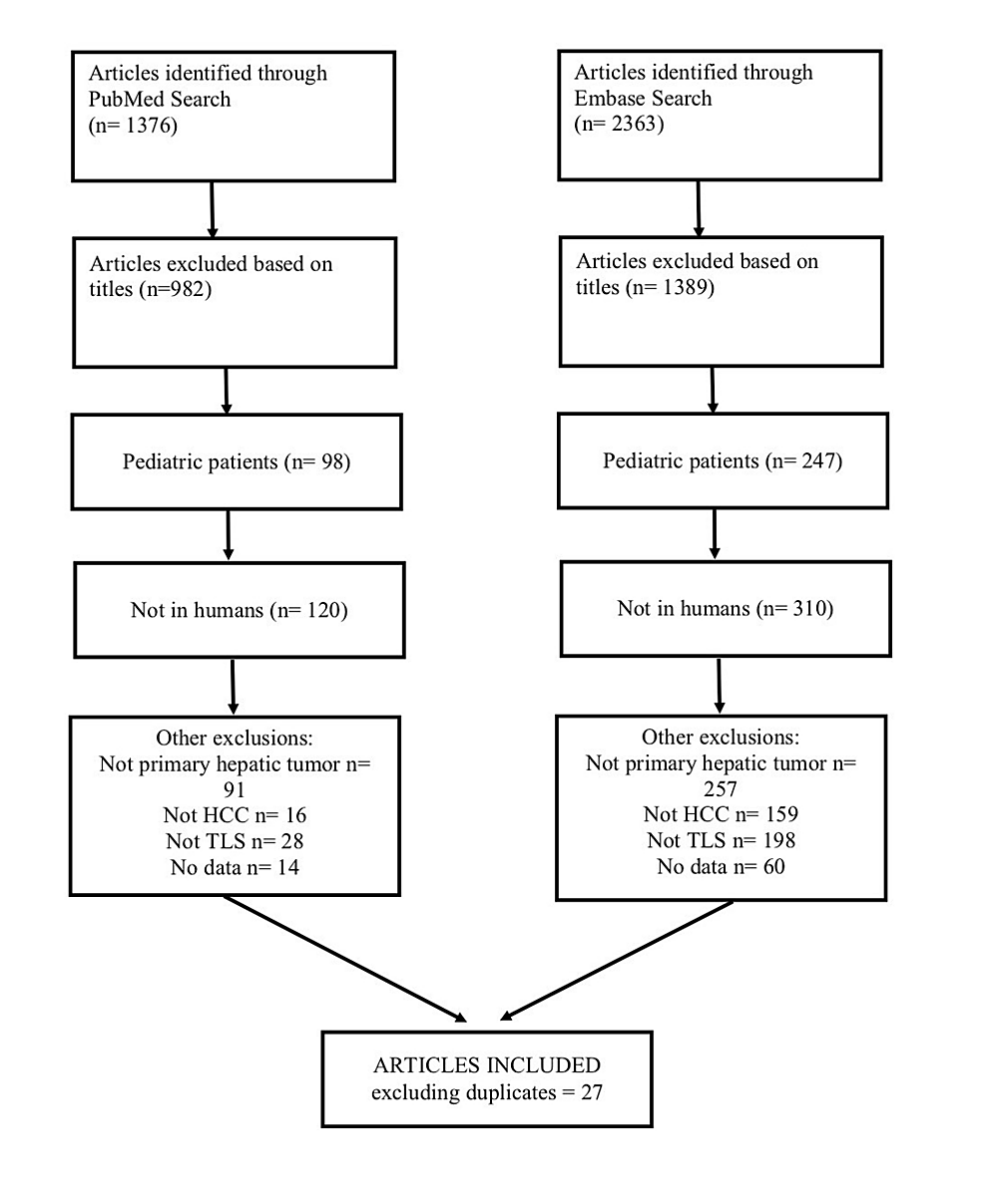
Flow diagram of literature search

We followed the guideline of Preferred Reporting Items for Systematic Reviews and Meta-Analyses (PRISMA) to conduct the present systematic review [4]. A comprehensive and current search for relevant articles was conducted in Medline (via PubMed) and Embase through May 2018. Searches of electronic databases were conducted both with controlled vocabulary (MeSH/Emtree) terms and free text terms (hepatocellular carcinoma OR HCC OR hepatic tumor OR liver malignancy OR liver OR hepatic OR liver carcinoma OR hepatic malignancy OR liver cancer AND tumor lysis syndrome). The search strategy is detailed in Figure 3.

Articles were included if they were in English and were full-text articles or abstracts. Review protocols were established, and paper copies were distributed to authors. Citations were assessed by two reviewers (TA and CCL), and discrepancies were resolved in consultation with a third reviewer (TJ). The quality assessments of individual studies were not feasible since all included studies were case reports. References of obtained articles and pertinent reviews were manually scanned for additional articles. We also included our own case in the cohort. The details of this case presentation are attached to the appendix.

Data were presented as median, interquartile range (IQR), and range for continuous variables and number of cases with percentages for categorical variables. We used the Mann-Whitney U test for analyzing continuous and ordinal variables. For categorical variables, we used the Chi-Square test for statistical analysis and Fisher's Exact test if the expected value in any of the cells was less than five. Patient demographics, tumor characteristics, attributable factors of TLS were recorded. Laboratory results were documented, such as alpha-fetoprotein (AFP), serum electrolyte levels, and kidney function. Change of serum creatinine level was defined as the difference between the highest and baseline serum creatinine levels on records. Other information in association with TLS such as the use of uric-acid lowering agents, time of onset of TLS following treatment, and mortality were recorded as well.

We attempted to report the Barcelona Clinic Liver Cancer (BCLC) staging system [5] of individual cases. If information on the BCLC stage was not readily available in published articles, we calculated or approximated the “at least BCLC stage” based on data provided in individual case reports. Two reviewers (TJ and CCL) assessed the results. Data entry was inputted manually into Microsoft Excel™ (Microsoft. Corporation, Redmond, Washington). For data analysis, we used SPSS Version 25.0 (released 2017, IBM SPSS Statistics for Windows. Armonk, New York: IBM Corp) was used for data analysis.

Results

The comprehensive search identified 28 cases of TLS in patients with HCC. Details of individual cases are listed in Table 1. 

**Table 1:** Demographics of all enrolled cases TLS: tumor lysis syndrome; TACE: Transcatheter arterial chemoembolization; NS: not specified; RFA: radiofrequency ablation

Author	Age	Gender	Treatment	Time of onset (days after treatment)	Uric-acid reducing agent	Survival
Burney et al., 1998 [5]	44	male	TACE	0.33	hydration	expired
Burney et al., 1998 [5]	46	male	TACE	NS	none	survived
Vaisban et al., 2003 [6]	72	male	spontaneous	NS	allopurinol	expired
Lehner et al., 2005 [7]	64	male	RFA	2	none	expired
Lee et al., 2006 [8]	62	male	thalidomide	15	none	expired
Sakamoto et al., 2007 [9]	55	male	TACE	2	none	expired
Shiba et al., 2008 [10]	77	male	TACE	3	allopurinol	survived
Huang et al., 2009 [11]	55	male	sorafenib	30	allopurinol	expired
Hsieh et al., 2009 [12]	76	female	TACE	3	allopurinol	expired
Hsieh et al., 2009 [12]	56	male	TACE	NS	allopurinol	survived
Joshita et al., 2010 [13]	33	male	sorafenib	4	none	survived
Wang et al., 2010 [14]	54	female	TACE	5	none	survived
Shiozawa et al., 2010 [15]	60	male	sorafenib	6	rasburicase	expired
Abbass et al., 2011 [16]	62	male	sorafenib	7	none	expired
Chao et al., 2012 [17]	51	male	TACE	0	rasburicase	survived
Kekre et al., 2012 [18]	76	male	spontaneous	NS	allopurinol	expired
Katiman et al., 2012 [19]	55	male	TACE	2	allopurinol	expired
Tsai et al., 2011 [20]	51	male	TACE	2	rasburicase	expired
Nishida et al., 2013 [21]	70	male	TACE	3	none	survived
Habib et al., 2013 [22]	59	male	sorafenib	21	none	expired
Mehrzad et al., 2014 [23]	70	male	none	NS	none	expired
Kudo et al., 2014 [24]	70	male	sorafenib	7	none	survived
Kim et al., 2015 [25]	55	male	sorafenib	8	allopurinol	expired
Kim et al., 2015 [26]	90	male	prednisolone	2	hydration	expired
Jiang et al., 2016 [27]	52	male	TACE	1	allopurinol	expired
Argawala et al., 2017 [28]	26	female	spontaneous	NS	none	expired
Imam et al., 2018 [29]	49	male	sorafenib	7	rasburicase	expired
Chou et al. [case vignette presented in this article]	42	male	sorafenib	8	febuxostat	expired

Patient demographics, attributable factors of TLS, and tumor characteristics of enrolled cases are summarized in Table 2.

**Table 2:** Patient demographics, attributable factors of TLS, and tumor characteristics of 28 cases TLS: tumor lysis syndrome; IQR: interquartile range; TACE: Transcatheter arterial chemoembolization; AFP: Alpha-fetoprotein; BCLC: Barcelona Clinic Liver Cancer *At least stage: the “at least stage” indicates that the BCLC stage of hepatocellular carcinoma was calculated or approximated using the available data in each case report

Variables	Median (IQR) or number of cases (%), range: minimum to maximum value
Age (years)	55.5 (51-70), range: 26 to 90
Sex (female)	3 (9.7)
Attributable factors	
TACE	12 (42.9)
Sorafenib	9 (32.1)
Spontaneous	3 (10.7)
Radiofrequency Ablation	1 (3.6)
Thalidomide	1 (3.6)
Prednisolone	1 (3.6)
Number of tumors	
Multiple	13 (46.4)
Single	12 (42.9)
Not specified	3 (10.7)
Length of the diameter of largest tumor	12 (7.6-16.8), range: 3 to 21, available in 24 cases
AFP (ng/ml)	15238 (2098-55625), range: 5 to 140700, available in 22 cases
BCLC staging	
*At least stage A	7 (25)
*At least stage B	10 (35.7)
*At least stage C	9 (32.1)
Stage B	1 (3.6)
Stage C	1 (3.6)

The median age of all cases was 55.5 years (IQR: 51-70 years). Three cases were female while the rest were male. In terms of attributable factors to TLS, transcatheter arterial chemoembolization (TACE) was the most common identified cause (12 cases, 42.9 %) followed by sorafenib therapy (nine cases, 32.1%). Regarding tumor characteristics, the median diameter of the largest tumor was 12 cm (IQR:7.6-16.8 cm, data available in 24 of 28 cases). Twelve cases (42.9%) had a single tumor in the liver, 13 cases (46.4%) had multiple tumors in the liver, and five cases (10.7%) did not specify the number(s) of tumors. The median AFP level was 1523.8 ng/ml (IQR: 2098-55625 ng/ml, data available in 22 of 28 cases). In regards to the BCLC staging of HCC, seven cases (25%) were at least BCLC stage A, 10 cases (35.7%) were at least stage B, and nine cases (32.1%) were at least stage C. Only two cases documented the actual BCLC staging in their articles: stage B (3.6%) and stage C (3.6%), respectively. The overall mortality of the cohort was 67.9%. Table 3 summarizes the time of onset of TLS, laboratory data, use of uric acid-lowering agents, and mortality of cases in our cohort.

**Table 3:** Time of onset, laboratory data, management, and clinical outcome of 28 cases of tumor lysis syndrome associated with hepatocellular carcinoma TLS: tumor lysis syndrome; IQR: interquartile range; HD: hemodialysis

Variables	Median (IQR) or number of cases (%)	Range: minimal to maximal value	Number of cases with available data
Time of onset of TLS following treatment (days)	3.5 (2-7.3)	Few hours to 30 days	23/28
Lab data during TLS			
Change of serum creatinine from baseline (mg/dl) to highest level (mg/dl) during TLS	3.0 (1.7-5.8)	-0.01 to 7.2	19/28
Potassium (mg/dl)	5.6 (5-6.2)	4.4 to 7.9	23/28
Uric acid (mg/dl)	12.2 (10.6-16.5)	4.4 to 22.9	28/28
Phosphorus (mg/dl)	6.9 (5.7-10.3)	3.9 to 16.7	20/28
Calcium (mg/dl)	7.3 (6.6-8.2)	2.3 to 14.6	19/28
Uric acid-lowering agents used during TLS			
Allopurinol	9 (32.1)		
Rasburicase	4 (14.3)		
Feboxustat	1 (3.6)		
Direct HD	4 (14.3)		
Not specified	10 (35.7)		
Outcome			
Death	19 (67.9)		
Survive	9 (32.1)		

The median time of onset of TLS following treatment was 3.5 days (IQR: 2-7.3 days, data available in 23 of 28 cases). The median difference between highest and baseline serum creatinine levels was 3.0 mg/dl (IQR:1.7-5.8 mg/dl, available in 19 of 28 cases). Of the 28 cases, 14 cases documented the use of uric acid-lowering agents. Of those, nine cases (32.1%) used allopurinol, four cases (14.3%) used rasburicase, one case (3.6%) used febuxostat, four cases (14.3%) received hemodialysis directly, and 10 cases (35.7%) did not specify any use of uric acid-lowering agents. 

In addition, we specifically compared patients who had TLS following TACE and those who had TLS after sorafenib therapy (Table 4).

**Table 4:** Comparison of patients’ demographics and clinical performance between the TACE group and the sorafenib group TACE: Transcatheter arterial chemoembolization; IQR: interquartile range; BCLC staging: Barcelona clinic liver cancer staging; TLS: Tumor lysis syndrome * At least stage: the BCLC stage of HCC was calculated or approximated using the available data in each case report

	TACE group median (IQR) or number of cases (%)	Sorafenib group median (IQR) or number of cases (%)	p-value
Age (years)	54.5 (51-66.5)	55 (45.5-61)	0.92
BCLC staging			
*At least stage A	5 (41.7)	0	0.002
*At least stage B	6 (50)	2 (22.2)	
*At least stage C	1 (8.3)	6 (66.6)	
Stage C	0	1 (11.1)	
Time of onset of TLS following treatment (days)	2 (0.8-3.0)	7 (6.5-14.5)	< 0.001
Mortality	5 (41.7)	7 (77.8)	0.18

Patients’ age was similar between the two groups. The median time of onset of TLS in the TACE group was significantly shorter than those in the sorafenib group (two days versus seven days, p < 0.001). Patients in the sorafenib group had a more advanced stage of HCC than those in the TACE group (p = 0.002). There was a trend toward increased mortality of patients in the sorafenib group in comparison with those in the TACE group (77.8% versus 41.7%, p = 0.18).

Discussion

The present review collects and summarizes published case reports of TLS in association with HCC. Similar to TLS in other solid tumors, TLS in HCC mostly occurs in the context of preceding therapies, such as TACE, sorafenib, or other types of chemotherapy. Though TLS in HCC is unambiguously characterized by massive cell death leading to metabolic derangements, the pathophysiology may vary when it comes to different attributable factors of TLS. TACE accounts for nearly half of reported TLS in HCC. It directly disrupts blood flow to tumor cells and leads to the massive destruction of tumor cells in a short time [19]. Sorafenib, on the other hand, interrupts the replication of tumor cells and angiogenesis without causing a direct cytotoxic effect [29]. While the exact mechanism of TLS following sorafenib therapy is obscure and less studied, we propose that rapid cell replication might be the predisposing factor of TLS in HCC following sorafenib treatment. The growth rate of HCC is determined by multiple cytogenetic factors such as the type of viral infection, genetic expression of HCC, and the interaction between tumor cells and surrounding hepatocytes [30]. Angiogenesis and nutritional supply to tumors are also environmental factors essential for tumor growth. Necrosis of bulky tumors may occur if the rate of angiogenesis cannot meet the nutritional demand of rapid cell division [31]. If angiogenesis is profoundly compromised following sorafenib treatment, the mismatch of blood supply and tumor growth can possibly occur and result in TLS. In some rare cases, this phenomenon occurs spontaneously if the tumor microenvironment fails to support tumor growth. Takeda et al. reported spontaneous necrosis of HCC in their patient and brought out a similar hypothesis in which spontaneous necrosis of HCC results from a sudden enlargement in the tumor, a reduced blood supply, and damage to the cancer nodule due to inflammatory cells [32].

Unsurprisingly, considering the different pathophysiology of TLS, we noticed patients who received TACE had a different clinical trajectory from those who received sorafenib therapy. The median time of onset of TLS was two days in the TACE group and seven days in the sorafenib group (p < 0.001). This could be explained by the different mechanisms of therapeutic modalities that were explained in the previous paragraph. More advanced stage of HCC was noted in the sorafenib group as compared to that of the TACE group (p=0.002). The mortality of TLS was 41.7% in the TACE group and 77.8% in the sorafenib group. While the sample size may be too small to show a significant difference between the two groups, the distinction in mortality rate may result from the difference in patients’ tumor stages.

There are several classifications and validated criteria for TLS. The most widely used definition of TLS is the criteria proposed by Cairo and Bishop in 2004 [2]. The establishment of diagnosis is either through laboratory abnormalities or by clinical presentations including seizure, cardiac arrhythmia, and death. In the majority of case reports in our study, the specific criteria used by authors were not documented although the laboratory criteria of TLS were likely met. We also noticed the diagnosis of TLS was delayed in many cases due to a number of reasons. First, TLS in HCC could have a vast array of symptoms such as dyspnea [6,25], abdominal pain [6], symptoms related to electrolyte imbalance (i.e. tetany) [27,28], and oliguria [5, 7-9]. This wide range of presentations was primarily affected by patients’ underlying diseases and could complicate the diagnostic process. Second, AKI and electrolyte imbalance resulting from other etiologies was a common finding in patients with cirrhosis or HCC [33]. The pathophysiology of AKI could be secondary to intravascular volume depletion, hepatorenal syndromes, drug toxicities, or other factors that affect renal perfusion [33]. Therefore, TLS typically was not the initial tentative diagnosis of AKI. Last, TLS due to sorafenib therapy may not be easily diagnosed since the median time of TLS following sorafenib therapy was seven days. It is worth mentioning that laboratory tumor lysis syndrome proposed by Cairo and Bishop [2] must occur within seven days of chemotherapy. Therefore, some cases with TLS due to sorafenib therapy may not meet the laboratory criteria and be underdiagnosed.

Uric acid-lowering agents are crucial in both the prevention and treatment of TLS. In prevention, these agents are used to avoid the precipitation of uric acid crystals in renal tubules during TLS. They are usually administrated prior to chemotherapy in patients with hematologic malignancies. For patients at risk of developing TLS, current guidelines recommend using allopurinol in patients with low to intermediate risk and rasburicase in patients with high risk for TLS prevention [34]. The indication of febuxostat was unclear and not mentioned in the current guidelines. In one randomized controlled trial, febuxostat was similar in safety and efficacy to allopurinol in preventing TLS and had been shown to be more effective than allopurinol in reducing serum uric acid levels [35]. In terms of treatment of TLS, allopurinol was once the mainstay of treatment. However, studies have shown rasburicase to be superior to allopurinol in treating TLS regarding the time of onset and effectiveness in decreasing serum uric acid levels [36]. According to the most updated guideline, rasburicase is the sole drug of choice for lowering uric acid levels in patients with an established diagnosis of TLS [34]. In cases that were included in our review, uric acid-lowering agents should be used for treatment purposes since they were administrated after the diagnosis of TLS was made. Therefore, rasburicase would be the preferred drug of choice for lowering uric acid levels. The predominant use of allopurinol in our included cases might be due to cost concerns and the limited availability of rasburicase in different healthcare systems.

The strength of our study is that it is the first systematic review of TLS in HCC and provides the most updated information on this topic. On the other hand, this study does have several limitations. First, since the available data is limited to case reports, no advanced statistical analysis can be conducted. Causation cannot be determined because of time sequencing and limitations associated with the type of study. Second, the quality of individual cases varies. Publication bias, which is selective revealing or suppression of information, certainly exists in case reports. This bias can be amplified in a systematic review with a larger collection of case reports. Third, except for one case report documenting the actual BCLC staging of HCC, all BCLC staging of included cases had to be either calculated or approximated based on the descriptions and data available in the articles. This limitation may affect the validity of the present view in summarizing tumor characteristics. Last, initial presentations and time of obtaining repeated blood tests were not clearly elaborated in many cases. Thus, to explicitly understand the disease course of TLS in HCC, further well-designed studies with universal protocols are needed.

## Conclusions

TLS rarely occurs in HCC but carries significantly higher mortality compared to TLS in hematologic malignancies. It may occur shortly after TACE or with a delayed onset following sorafenib therapy. TLS in HCC can present with a variety of symptoms and establishment of diagnosis is often delayed. TLS with different attributed factors can have distinct disease trajectories and clinical outcomes. Considering the kaleidoscope of novel therapies and diverse pathogenesis of HCC, it has become crucial for clinicians to recognize the clinicolaboratory derangements suggestive of TLS and initiate appropriate management.
